# Anion Complexation by an Azocalix[4]arene Derivative and the Scope of Its Fluoride Complex Salt to Capture CO_2_ from the Air

**DOI:** 10.3390/molecules28166029

**Published:** 2023-08-12

**Authors:** Angela F. Danil de Namor, Nawal Al Hakawati

**Affiliations:** 1 Laboratory of Thermochemistry, School of Chemistry and Chemical Engineering, University of Surrey, Guildford GU2 7XH, UK; 2 Department of Biological Sciences, Faculty of Science, Beirut Arab University, Tripoli 1300, Lebanon; n.elhakawati@bau.edu.lb

**Keywords:** azocalix[4]arene, fluoride complex salt, carbon dioxide capture

## Abstract

A newly synthesized upper rim azocalix[4]arene, namely 5,11,17,23-tetra[(4-ethylacetoxyphenyl) (azo)]calix[4]arene, CA-AZ has been fully characterized, and its chromogenic and selective properties for anions are reported. Among univalent anions, the receptor is selective for the fluoride anion, and its mode of interaction in solution is discussed. The kinetics of the complexation process were found to be very fast as reflected in the immediate colour change observed with a naked eye resulting from the receptor–anion interaction. An emphasis is made about the relevance in selecting a solvent in which the formulation of the process is representative of the events taking place in the solution. The composition of the fluoride complex investigated using UV/VIS spectrophotometry, conductance measurements and titration calorimetry was 1:1, and the thermodynamics of complexation of anions and CA-AZ in DMSO were determined. The fluoride complex salt was isolated, and a detailed investigation was carried out to assess its ability to remove CO_2_ from the air. The recycling of the complex was easily achieved. Final conclusions are given.

## 1. Introduction

Carbon dioxide among all gases has been an issue of extensive political debate due to its importance as a greenhouse gas [1,2,3] which is a key cause of global warming. In addition to its environmental impact, the gas has been employed in the chemical industry, food, modern agriculture and medical diagnosis due to its vital role in human physiology. Hence, the development of new methodology for the selective, quantitative and rapid recognition of this inert gas [4,5,6,7] is a matter of priority concern. Methods such as gas chromatography, infrared spectroscopy and electrochemistry (Severing Haus electrodes) are used to detect CO_2_. More recently, few attempts have been made to develop optical chemo sensors based on UV spectroscopy, colorimetry or fluorescence to sense carbon dioxide such as those based on α-cyanostilbene [8] or europium hexafluoroacetylacetonate optical sensor [9] for sensing carbon dioxide in solution or a pyrene-based chemosensor for carbon dioxide gas [10]. Interestingly, few studies have shown that chemo sensor compounds displaying high selectivity for the fluoride anion were able to detect carbon dioxide gas with full restoration of their original colours and spectral properties [11,12]. Xia and co-workers reported the carboxylation of pyrimido [1,2] benzimidazole and squarine derivatives by the aid of carbon dioxide in the presence of an excess of a fluoride salt [Bu_4_N] F [13,14]. 

Encouraged by the single step procedure introduced by Gutsche [15] for the synthesis of parent calix[n]arenes, for several years we have been focused on the thermodynamic aspects of calixarene chemistry [16], mainly narrow rim functionalized calixarenes [17,18,19,20]. Recently, we have proceeded with an investigation on wide rim calixarene derivatives involving azo functionalities. The design of chromoionophores with multifunctional sensor systems attracted much attention due to their usefulness in the development of chemical sensors [21]. These sensors exhibit colour changes as they interact with ionic or molecular species [22,23] which can be easily detected with the naked eye without the use of any spectroscopic technique. More particularly, azophenol-based chemo-sensors have been widely incorporated into the chromoionophores. These compounds can display a change in their absorption spectra in addition to a colour change upon interaction with metal cations [24,25,26,27,28,29] or anions [30,31,32,33,34,35,36,37,38,39,40,41]. A series of non-macrocyclic azophenol derivatives as chromogenic units designed to selectively generate colour change as they complex with different anions has been reported by Hong and co-workers [30,31,32,33,34,35,36,37,38,39,40,41].

The introduction of the azo functionality in macrocyclic compounds has been reviewed by Wagner-Wysiecka and co-workers in 2018 [42]. Much attention has been devoted to the development of fast sensors for analysing fluoride due to the anion dual functionality, whether in the treatment of osteoporosis and prevention of dental decay, fluorosis or other bone diseases like osteosarcoma [43,44,45,46]. 

Most of the work carried out in the field of supramolecular chemistry has been mainly concerned with complexation studies involving ionic and neutral species with little attention on the properties of these new complex salts and their applications. CO_2_ fixation has been elegantly investigated in solution and in the solid state by Bazzicalupi and coworkers [47] using copper (II) and zinc (II) complexes of [15]aneN_3_O_2_ macrocycles.

Upper rim functionalized calix[4]arenes have been attached to silica for the production of selective materials for CO_2_ [48]. However, there are no reports on CO_2_ uptake by anion complex salts of calix[4]arene in solution and in the air. Hence, we report here an easily recyclable chemo-sensor probe for the detection and capture of CO_2_ in air, based on the tetra-n-butyl ammonium *para*-ester diazo phenyl calix[4]arene fluoride salt, Bu_4_N [CA-AZ F]. In doing so, fundamental studies on the synthesis and characterization of the free receptor, *para*-ester diazo phenyl calix[4]arene, CA-AZ and its complexing properties in solution are also included.

## 2. Results and Discussion

### 2.1. Structural Characterization of 5,11,17,23-tetra[(4-ethylacetoxyphenyl) (azo)]calix[4]arene (CA-AZ)

#### 2.1.1. ^1^H NMR Characterization of CA-AZ in DMSO-d_6_ and CDCl_3_ at 298 K

^1^H NMR analyses of CA-AZ in DMSO-d_6_ and CDCl_3_ were done to identify its conformational morphism and the changes that it undergoes upon interaction with guest species (Table 1). Gutsche [16] has stated that the difference in the chemical shifts (Δδ_ax-eq_, ppm) between the low and high field pairs of resonances arising from the methylene protons (H-equatorial H-3 and axial H-3′) of the calix[4]-based receptor provides a measure of the ‘flattening’ of the ‘cone’. Thus, for a ligand in a perfect ‘cone’ conformation, the Δδ_ax-eq_ = 0.90 ppm and decreases significantly in the ‘flattened’ conformation or increases when the aromatic rings become more parallel to each other and the macrocyclic adopts a distorted ‘cone’ conformation. Considering the above statements, the almost ‘cone’ conformational morphism of CA-AZ was detected in DMSO-d_6_ where the ∆δ_ax-eq_ = 0.83 ppm. In CDCl_3_, a flattened ‘cone‘ conformation with Δδ = 0.53 ppm is found. Additionally, it should be mentioned that the ligand exhibited a red colour in DMSO-d_6_. 

#### 2.1.2. FT-ATR Characterisation of Para-Ester Diazophenylcalix[4]arene

**Figure 1:**
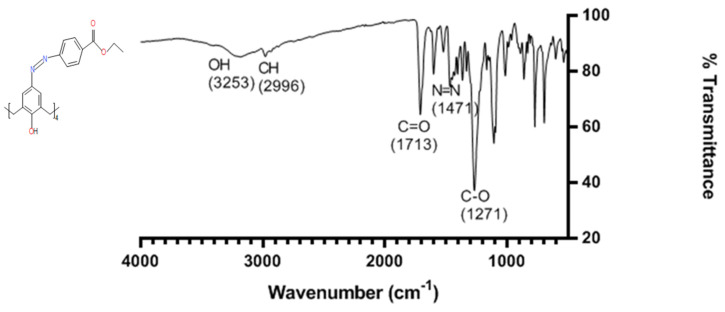
Infrared spectrum of CA-AZ displaying the most important bands of the ligand.

The infrared spectrum of CA-AZ is shown in Figure 1. The broad band at 3221 cm^−1^ is assigned to the -OH stretching vibrations of the phenol ring which is within the 3100–3500 cm^−1^ range found for all parent calixarenes [49]. The lower position of the OH stretch vibration may be attributed to hydrogen bond formation in the narrow rim as previously found for these macrocycles [49]. The strong band at 1713 cm^−1^ in the spectrum of CA-AZ corresponds to the C=O stretching vibration [50]. The N=N stretching mode appears in the region of 1467 cm^−1^ [50]. The weak band at 1362 cm^−1^ is attributed to the aryl group [50]. The C-O stretching mode is represented by an intense band at 1266 cm^−1^.

#### 2.1.3. Thermal Stability of *Para*-ester diazophenylcalix[4]arene

**Figure 2:**
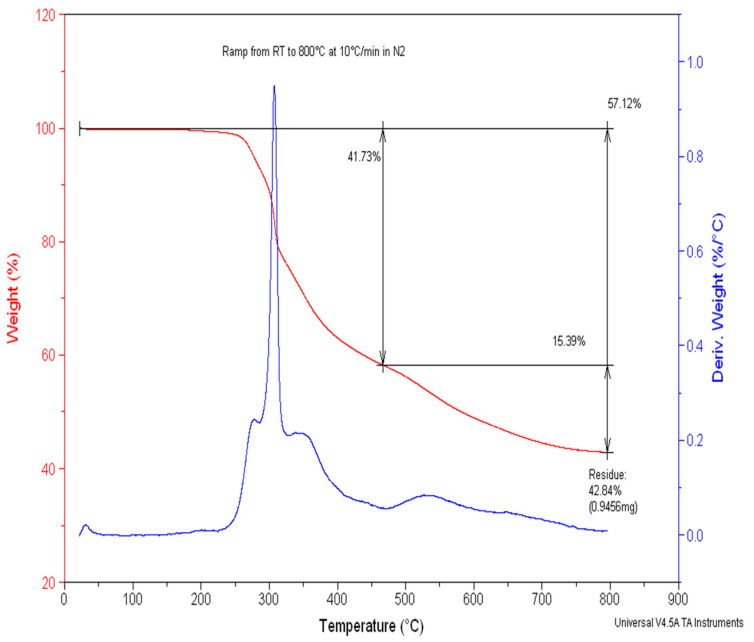
The TG curve of CA-AZ.

An important parameter that should be determined is the thermal stability of the ligand as it is relevant for its applications. This was investigated through thermogravimetric analysis where the TG curve is presented in Figure 2. The curve below shows that the ligand can preserve thermal stability up to 280 °C. Two exothermic phases are shown in *para*-ester diazophenylcalix[4]arene TG curve. The first one corresponds to the release of moisture from the surface (average experimental mass loss of 15.39%) followed by the second one indicating the decomposition of the whole compound with an average mass loss of 57.12%.

### 2.2. ^1^H NMR Complexation of CA-AZ with Anions in DMSO-d_6_ at 298 K

DMSO was the selected solvent for further experimental work, given that in chloroform, anion salts are predominantly as ion pairs.

Screening experiments were conducted to determine the active sites of interaction of CA-AZ with different ionic species (cations and anions). Based on the ^1^H NMR findings, the ligand showed no interactions with alkali, alkaline-earth and transition metal ions where no chemical shift changes were observed. Chemical shift values (δ in ppm) of CA-AZ with F^−^, H_2_PO_4_^−^, HSO_4_^−^ and Cl^−^ as tetra-n-butyl ammonium salts and CO_3_^2−^ as ammonium salt are all listed along with the free ligand in Table 2. Furthermore, colour changes from faint red to dark purple and dark red were observed immediately after the addition of F^−^ and H_2_PO_4_^−^ salts (1 × 10^−3^ mol·dm^−3^), respectively, to CA-AZ.

As shown in Table 2, some protons (H-2, H-4, H-4′ and H-5) showed severe broadness to the extent that they did not show up in the spectrum upon the addition of F^−^. Quite clearly from the Δδ_ax-eq_ values of the free and complex CA-AZ, the addition of the F^−^ salt produces a dramatic conformational change in which the ligand adopts a ‘flattened’ cone conformation suggesting that the intramolecular hydrogen bonding between the hydroxyl group(-OH) in the narrow rim of the free receptor is broken as a result of the receptor–anion interaction. 

The conformational changes that CA-AZ undergoes with H_2_PO_4_^−^ could not be calculated due to the broadening of the equatorial (H-3) signal. As far as HSO_4_^−^ is concerned, the change is significant with the macrocycle adopting a ‘distorted’ ‘cone’ as reflected in the Δδ_ax-eq_ value of 1.57 ppm. This is in contrast with the Δδ_ax-eq_ of 0.70 ppm found for the CA-AZ-Cl^−^ where the macrocycle adopts a slightly ‘flattened’ conformation much less pronounced than that observed for the fluoride complex. Deshielding of the equatorial and axial protons (H-3 and H-3′) changed the Δδ_ax-eq_ value from 0.83 ppm to 0.99 ppm as a result of the CA-AZ- CO_3_^2−^ interaction.

Up-field shifts in the aromatic protons (H-2 and H-4) of CA-AZ were observed upon complexation with H_2_PO_4_^−^, while those for CA-AZ-HSO_4_^−^ and CA-AZ-Cl^−^ complexes remained unchanged. Up-field shifts in H-6 (methylene proton) of the same magnitude were detected for the three complexes except for CO_3_^2−^ (ammonium counter-ion). Three signals for tetra-n-butyl ammonium counter ion were shown. Interestingly, the intensities of tetra-n-butyl ammonium peaks in the spectrum of CA-AZ-F^−^ complex were lower than those involving other anions. The same finding of H-6 chemical shift was obtained but with different counter ion. ^1^H NMR complexation analysis of CA-AZ with NaF in DMSO-d_6_ was unclear due to the very low solubility of the salt in DMSO-d_6_. Up-field shift of the aromatic protons (H-2 and H-4) was observed in the ^1^H NMR spectrum of CA-AZ-CO_3_^2−^ complex. Moreover, these peaks became broad as soon as an interaction between CA-AZ and CO_3_^2−^ through hydrogen-bonding formation took place. It is expected that the aromatic peaks will continue to broaden with further addition of CO_3_^2−^ until they disappear. The interaction of CO_3_^2−^ with CA-AZ yielded a slight change in the ligand conformation. No changes in the chemical shifts of the ligand were observed from ^1^H NMR studies of CA-AZ and NaNO_3_ in DMSO-d_6_. 

### 2.3. Chromogenic Properties and UV-VIS Experiments

CA-AZ possesses dual functionality for its selectivity for a given ion through the phenolic hydroxyl binding sites and its sensitivity as a chemo-sensor with covalently linked azo moieties. 

**Figure 3:**
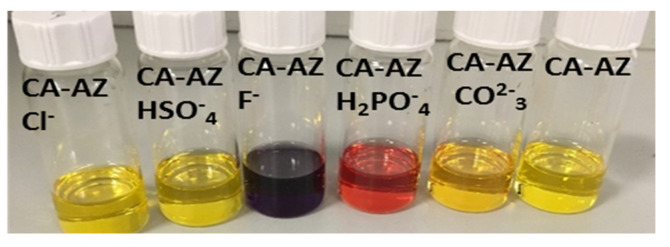
Colour changes of CA-AZ resulting from anion interactions in DMSO.

**Figure 4:**
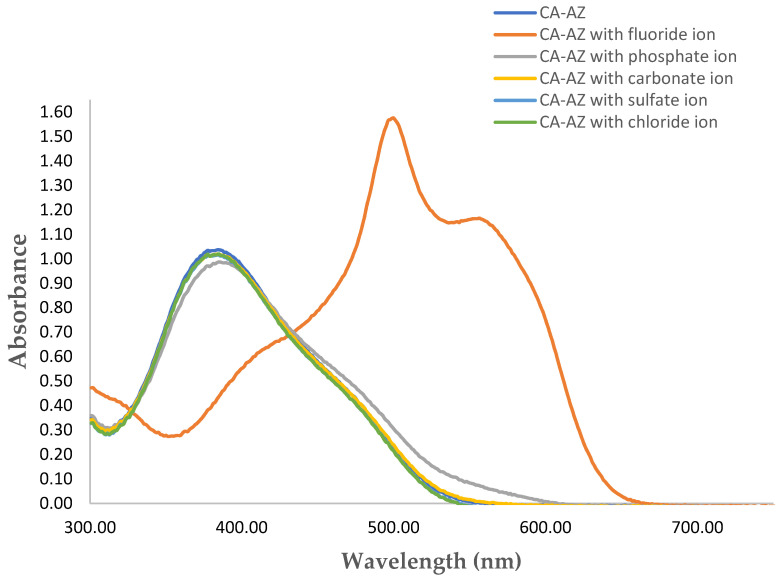
Spectral changes of *para*-ester diazophenylcalix[4]arene (1 × 10^−5^ mol·dm^−3^) with different anions.

The sensitivity function was determined from the chromogenic properties of the ligand upon its complexation with different ionic species in DMSO (Figure 3) along with the spectral changes in the UV-VIS analysis (Figure 4). Colour changes from yellow to purple and red were detected immediately after the addition of F^−^ and H_2_PO_4_^−^ (1 × 10^−4^ mol·dm^−3^ as tetra-n-butyl ammonium salt) to CA-AZ (1 × 10^−5^ mol·dm^−3^) in DMSO. However, the addition of higher concentrations of these anion salts turned the colour even darker (dark blue in the case of F^−^ and maroon red with H_2_PO_4_^−^). Moreover, a change in the colour from yellow to orange was observed with CO_3_^2−^ (ammonium counter-ion). No visible changes in the colour of CA-AZ were detected with addition of HSO_4_^−^ and Cl^−^ (Figure 4). 

Several studies reported two possible tautomeric forms of azocalix[4]arenes [51,52,53,54], azophenol and quinone-hydrazone through which they possess different visible spectral features, and this is also dependant on the substituents of the azocalix[4]arene derivatives [55]. In the case of CA-AZ, the absorption maxima (λmax) in the UV-spectrum are observed at 384 nm which corresponds to a π-π* transition suggesting that the ligand exists in the azophenol form [56]. However, visible spectral changes of CA-AZ were observed with the addition of F^−^ and H_2_PO_4_^−^ from 384 nm to 501 nm with shoulders at 561 and 393 nm, respectively. A marked bathochromic shift is found for F^−^ with an isosbestic point at 433 nm. This spectral change corresponds to a n-π* transition indicating the presence of CA-AZ in a quinone-hydrazone tautomeric form. Chawla and Gupta [57] suggested a charge transfer induced to the acidic ligand protons via the addition of basic F^−^salt. Moreover, azophenol chromophore electron excitation occurs through charge transfer from the phenol (oxygen donor atoms) to the substituents of the chromophore unit (acceptor atoms) which results in a colour change [58]. Chen and co-workers [59] reported similar findings with calix[4]arene derivatives for anion chromogenic sensors. Thiampanya and co-workers [60] reported a charge transfer in an azocalix[4]arene strapped calix[4]pyrrole from the donor oxygen atom of the azophenol moiety to the acceptor atom in the chromophore substituent following addition of basic anions. The outcome of this investigation indicates that CA-AZ is more sensitive to F^−^ than the remaining anions, where a significant visible spectral shift was observed through the addition of this anion to the ligand. 

Given that the aim of this paper was to isolate the fluoride complex salt, we proceeded with ^1^H NMR titration of CA-AZ with the fluoride anion salt in DMSO-d_6_ as well as UV-Visible spectroscopy, conductance measurements and titration calorimetry to determine the composition of the fluoride complex salt and its stability. 

### 2.4. ^1^H NMR Titration of para-ester diazophenylcalix[4]arene with Fluoride Anion as Tetra-N-butyl Ammonium in DMSO-d_6_

**Figure 5:**
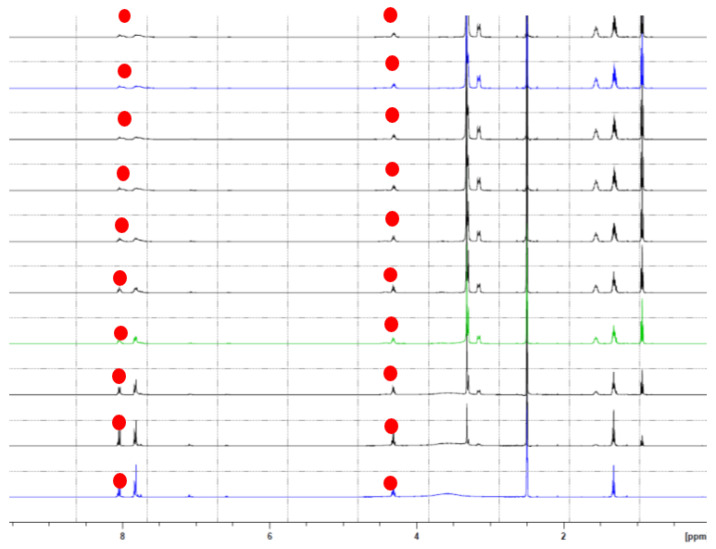
^1^H NMR spectral changes of CA-AZ titrated with an increased concentration of 18 × 10^−4^ mol·dm^−3^ of F^−^ at 298 K. Changes in the characteristic peaks of CA-AZ as a result of F^-^ addition are marked with red dots.

Knowing that CA-AZ exhibits sensitivity and selectivity for F^−^, ^1^H NMR titration of the ligand with the fluoride anion salt in DMSO-d_6_ was carried out to gain more insights on its binding mode. ^1^H NMR spectra in Figure 5 reveals the mode of interaction of CA-AZ with the anion. As the addition of TBAF (tetra-n-butyl ammonium fluoride) progresses, the peaks corresponding to the aromatic protons (H-2, H-4 and H-4′) and ethyl proton (H-5) along with the equatorial and axial protons became more broad until they almost disappeared but not shifted at all. From the ^1^H NMR titration outcomes, it becomes clear that further addition of fluoride to CA-AZ leads to a deprotonation of one of the phenolic OH functionality in the narrow rim of the receptor. This statement is in agreement with the findings by Zheng and co-workers [61]. These authors demonstrated that the addition of higher concentrations of F^−^ in the ^1^H NMR titration of a calix[4]arene derivative have resulted in the disappearance of the aromatic peaks and very small chemical shifts in the characteristic peaks of the receptor.

This mode of interaction between the ligand and F^−^ is highly dependent on the concentration of both in the medium. However, when the concentration of the ligand or the F^−^ varies, different interaction behaviour will be observed as in the case of ^1^H NMR complexation of CA-AZ and F^−^ where the fluoride salt was in excess of the receptor (Table 2).

### 2.5. UV-VIS Titration of CA-AZ with the Fluoride Anion Salt in DMSO

**Figure 6:**
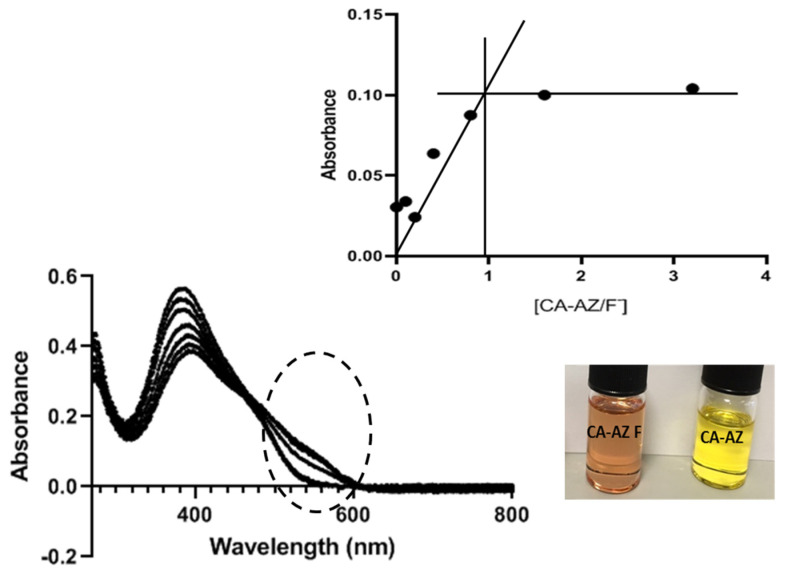
Plot of absorbance at 530 nm as a function of [CA-AZ]/[F^−^] resulting from the UV-VIS titration of CA-AZ with F^−^ in DMSO.

Figure 6 shows that as the concentration of fluoride increases, the absorbance increases until the [CA-AZ:F^−^] ratio is 1:1. Then, the absorbance remains constant. However, the addition of F^−^ turns the colour from yellow to faint pink which is not altered by the further addition of the fluoride salt.

### 2.6. Conductometric Studies of CA-AZ Interacting with Fluoride Anion in DMSO at 298.15 K

**Figure 7:**
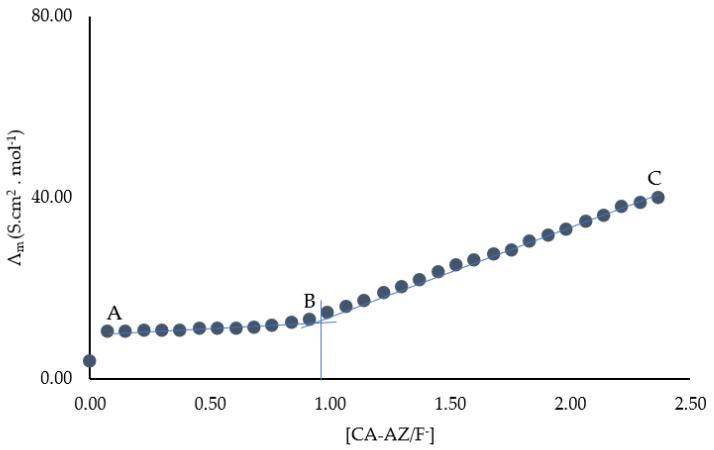
Conductometric titration of CA-AZ with F^−^ in DMSO at 298.15 K.

Conductometric titration experiments were carried out to have further evidence of the composition of the complex (Figure 7). There was a slight change in conductance from A to B due to the complexation with a significant increase from B to C due to the excess of fluoride ions in solution. A well-defined change in the curvature was observed at the reaction stoichiometry, suggesting the formation of a 1:1 complex. This outcome of conductance measurements agreed with that obtained using UV-VIS spectroscopy.

### 2.7. Thermodynamics of Complexation of CA-AZ with Fluoride in DMSO at 298.15 K

Despite the importance of thermodynamics in assessing quantitatively the selectivity of azo-calix[4]arenes for ionic species in a given medium, hardly any such study has been reported in the literature. Therefore, calorimetric titrations of CA-AZ with anions^−^ in DMSO were conducted. Thus, the stability constant (log K_s_), hence ($\Delta_{c}G^{o}$), enthalpy ($\Delta_{c\;}H^{o}$) and entropy ($\Delta_{c\;}S^{o}$) of the complexation are reported in Table 3. The data are referred to the process represented in Equation (1).

(1) $$X^{-}\;(\mathrm{DMSO})+\mathrm{CA}\text{-}\mathrm{AZ}\;(\mathrm{DMSO})\;\overset{\mathrm{Ks}}{\to }\;[\mathrm{CA}\text{-}\mathrm{AZ}\;X^{-}]\;(\mathrm{DMSO})\;$$

In Equation (1), [CA-AZ X^−^] denotes the anion complex. The data indicate that among the univalent anions, the receptor is selective for fluoride. The complex is rather stable in this solvent, which allows the isolation of the fluoride salt. Its stability results from the favourable contribution of both the enthalpy and the entropy, but the process is entropically controlled. The positive entropy is likely to be attributed to the de-solvation of the anion or the receptor, or both upon complexation. Although we used a competitive method to obtain the thermodynamic parameters associated with the complexation of CA-AZ and chloride in DMSO, as shown in Table 3, the heat observed is too small as to derive an accurate value for the stability of the chloride complex. No measurable heats were obtained for the complexation process involving CA-AZ and bromide and iodide anions in DMSO. The stability constant of the hydrogen sulphate complex with this receptor in DMSO is indicative of the formation of a weak complex. The binding process is entropy-driven given that the enthalpy does not make a favourable contribution to the stability of the complex. These data are typical of processes in which de-solvation of the anion or the receptor or both takes place upon complexation.

As far as bivalent anions are concerned, the processes taking place are as follows:

(2) $$X^{2-}\;(\mathrm{DMSO})+\mathrm{CA}\text{-}\mathrm{AZ}\;(\mathrm{DMSO})\;\overset{\mathrm{Ks}}{\to }\;[\mathrm{CA}\text{-}\mathrm{AZ}\;X^{2-}]\;(\mathrm{DMSO})\;$$

(3) $$\left[\mathrm{CA}\text{-}\mathrm{AZ}\;X^{2-}]\;(\mathrm{DMSO})+X^{2-}\;(\mathrm{DMSO})\;\overset{\mathrm{Ks}}{\to }\;[\mathrm{CA}\text{-}\mathrm{AZ}\;X^{4-}]\;(\mathrm{DMSO}\right)$$

The receptor shows a great affinity for the carbonate anion in DMSO and a greater hosting ability for this anion. Thus, the formation of the 1:1 complex is favoured by the enthalpy and entropy contributions, but it is enthalpy controlled. However, the formation of a 1:2 (receptor: anion) complex may lead to a high degree of disorder in the system due to the entrance of a second anion. This is reflected in the positive entropic contribution which controls the stability of the complex and overcomes the unfavourable enthalpic contribution. Given that the aim of this paper is to assess the ability of the fluoride salt to sense and capture CO_2_, the higher selectivity of the receptor for the carbonate anion relative to fluoride or any other anion is not an issue given that salts other than fluoride are unable to interact with this gas in the air. We were unable to determine the thermodynamics associated with the binding process involving hydrogen phosphate given that the composition of the complex could not be accurately established, and therefore, it is pointless to derive thermodynamic data for an undefined process

Having established the stability of the fluoride complex we proceeded with its isolation and characterization of the complex salt as described below.

### 2.8. SEM-EDX and FT-IR Characterization of Bu_4_N [CA-AZ F]

**Figure 8:**
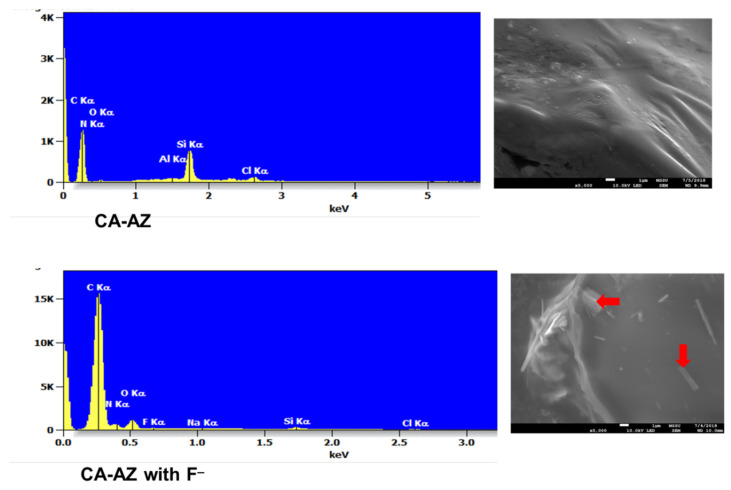
Morphological micrographs of azocalix[4]arene (CA-AZ) and azocalix[4]arene loaded with fluoride ion (red arrows) with corresponding EDX spectra showing the elemental composition. ×5000 original magnification, AI peak corresponds to aluminium stub used for samples’ mounting.

SEM provides the morphological characteristics of the compound whereas EDX generates its elemental composition. Inspection of the micrographs of CA-AZ and CA-AZ loaded with F^−^ (Figure 8) shows the presence of the ion (red arrows) in the compound. A change in the morphology of its surface is also found which is referred to the complexation of the compound with the fluoride ion. Regarding the EDX spectra, the characteristic peaks of the CA-AZ are shown, while for the CA-AZ-F^−^ salt spectrum, an additional peak for fluoride is present that is attributed to the presence of fluoride in the compound. The increase in the carbon peak is due to the presence of tetrabutylammonium salt.

**Figure 9:**
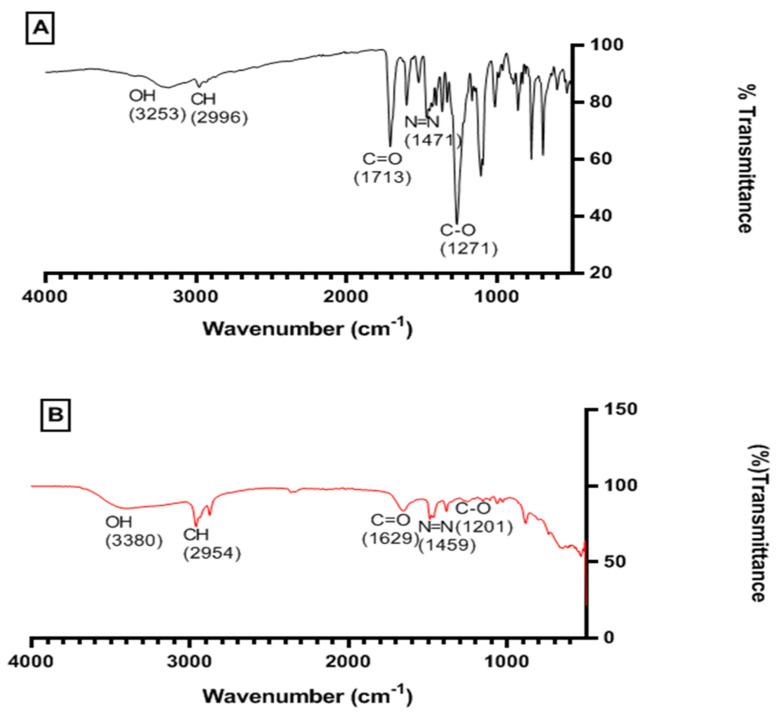
FTIR spectra of (**A**), azocalix[4]arene and (**B**), azocalix[4]arene loaded with TBAF.

The azocalix[4]arene and the fluoride complex salt also assessed via FTIR recorded in the range 4000–500 cm^−1^ are displayed in Figure 9. The characteristic peaks which are attributed to the phenol OH stretching are shown in both spectra (Figure 9A,B) as broad bands at 3253 cm^−1^ for the compound and 3380 cm^−1^ for fluoride complex salt. The bands at 2996 and 2954 cm^−1^ in A and B spectra are referred to C-H stretching vibration of CH_2_ in azocalix[4]arene and its fluoride complex salt. Inspecting the characteristic peak positions in both spectra, a blue shift of +127 cm^−1^ in the OH band is observed with a reduced intensity and a red shift of −42 cm^−1^ in C-H band with an increased intensity indicating an interaction between the compound and a fluoride ion. Additionally, the three bands at 1713, 1471 and 1271 cm^−1^ correspond to C=O, N=N and C-O vibration in azocalix[4]arene and are red shifted to 1629, 1459 and 1201 cm^−1^ with decreased intensity upon the interaction of the compound with the fluoride salt. Interestingly, the strong band at 1010 cm^−1^ in CA-AZ corresponding to the C-OH vibration almost disappeared from the complex salt spectrum due to a deprotonation of the compound caused by F^−^, leading to a blue shift in the OH band of the complex. 

### 2.9. Interaction of Bu_4_N[CA-AZ F] with CO_2_

**Figure 10:**
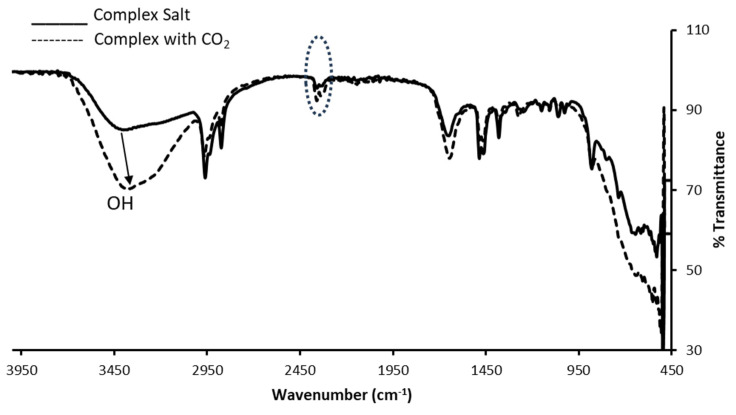
FTIR spectra of the complex salt and complex salt saturated with carbon dioxide.

The CA-AZ was designed to host and sense ionic species, but upon isolating the Bu_4_N[CA-AZ F] salt, it was discovered that while the free receptor does not interact with CO_2_, the fluoride salt does. It detects and captures CO_2_ from air, where the colour changes from red to orange yellow (Figure 10). The process of gas detection by the complex salt was established within seven seconds. These observations were further investigated by FTIR studies to explore the mode of interaction with CO_2_. 

The infrared spectrum of the solid complex salt is shown in Figure 10. Characteristic bands of the complex salt are explained in Section 2.8. However, the broad band at 3345 cm^−1^ which is assigned to the -OH stretching vibrations of the phenol ring in the solid complex was redshifted with an increased intensity and less broadened upon the complex exposure to CO_2_. Moreover, the band at 1675 cm^−1^, attributed to the C=O vibration, was redshifted to 1650 cm^−1^ as it got sharper upon the interaction of the complex salt with carbon dioxide. Inspecting the C=O band of the complex, the broadness of the signal was noticed when compared with the sample treated with CO_2_.. Hellwig [62] explained that the broadening band of the C=O results from its interaction with water. We are strongly of the view that unlike the free receptor, the fluoride complex salt is highly hygroscopic, and as such, the presence of water promotes the interaction of the fluoride complex salt with CO_2._ through hydrogen formation between the hydrogen atoms of water and the oxygen atoms of the loaded gas. No shifts were observed in the bands at 1457 and 1251 cm^−1^ which correspond to the N=N and the C-O stretching vibrations in the spectrum of the carbon dioxide-loaded probe. Moreover, the IR spectrum of the probe with carbon dioxide shows a peak at 2327 cm^−1^ which is attributed to the gas.

#### 2.9.1. Extraction of Carbon Dioxide by the Solid Complex Salt

**Figure 11:**
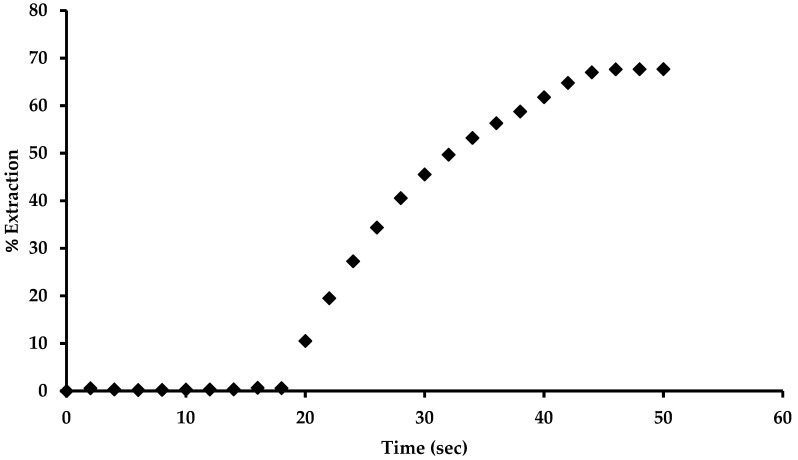
Plot showing of percentage of carbon dioxide extraction by the complex versus time (s).

The solid complex salt (Bu_4_N [CA-AZ F]) partially hydrated was nourished with CO_2_ gas in a fixed-bed reactor where the extraction percentage was recorded at different time intervals (Figure 11). Inset in Figure 11 shows an increase in the percentage removal of CO_2_ from the air which started after 18 s where it reached 68% after 44 s. As in any extraction process, the percentage of extraction is dependent on the experimental conditions selected.

Our aim was to show the ability of the receptor to sense and capture the gas, and this was successfully demonstrated. In this experiment, the partially hydrated salt was used to be able to determine exactly the weight of the material to be used in the reactor, and therefore, it was clear through naked eye detection that at the end of the experiment, the material did not develop the full colour observed for a fully hydrated salt saturated with CO_2_. 

#### 2.9.2. Recycling of the Complex

**Figure 12:**
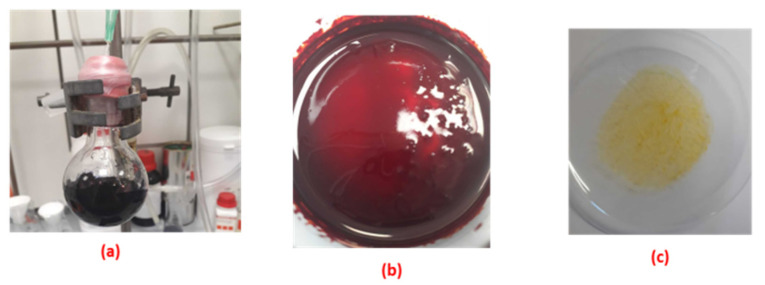
Recycled solid complex salt placed under vacuum (**a**); complex salt initially exposed to CO_2_ (**b**); complex salt fully exposed to CO_2_ (**c**).

Carbon dioxide was able to be removed from the complex salt when purged with nitrogen gas for three minutes or placed under vacuum where it retained its original colour (dark blue). The dark blue colour of the complex salt means that it had zero carbon dioxide, and this would not be observed unless it were placed under vacuum (Figure 12a). However, when the solid complex salt was exposed to carbon dioxide, the colour changed to red and then to yellow with more CO_2_ exposure, and no more colour change was observed, indicating the saturation of the solid complex salt with carbon dioxide (Figure 12). Interestingly, the yellow-coloured solid complex was recycled back to dark blue when it was placed under vacuum. 

## 3. Materials and Methods

### 3.1. Chemicals 

All chemicals used throughout the work were of analytical grade. Salts used throughout this study were placed in a vacuum oven and then stored in vacuum desiccators over phosphorus pentoxide, P_4_O_10_, for several days to remove water, before being used for experimental purposes. *p*-*tert*-Butyl calix[4]arene (C_44_H_56_O_4_, ≥ 97%), ethyl 4-aminobenzoate (H_2_NC_6_H_4_CO_2_C_2_H_5_, 98%), phenol (C_6_H_6_O, ≥99%), sodium ethanoate trihydrate (C_2_H_3_NaO_2_·3H_2_O, ≥99%), sodium nitrite (NaNO_2_, 97+ %), aluminium (III) chloride (AlCl_3_, 98.5%), tetra-n-butylammonium chloride ((C_4_H_9_)_4_NCl, ≥97.0%), tetra-n-butylammonium flouride hydrate ((C_4_H_9_)_4_NF·H_2_O), 98%), tetra-n-butylammonium dihydrogen phosphate ((C_4_H_9_)_4_NH_2_PO_4_)), 97%), tetra-n-butylammonium hydrogen sulfate ((C_4_H_9_)_4_NHSO_4_, 97%) and potassium carbonate (K_2_CO_3_, 99+ %) were purchased from Sigma Alrich.

Deuterated solvents used in NMR experiments, dimethyl sulfoxide-d_6_ (C_2_D_6_OS, 99.9%) and chloroform-d (CDCl_3_, 99.8% + 0.05% *v*/*v* TMS) were purchased from Cambridge Isotope Laboratories.

#### 3.1.1. Synthesis of 25, 26, 27, 28-Tertrahydroxy Calix[4]arene via de-Tert-Butylation (Friedel Craft de-Tert-Butylation Reaction)

De-*tert*-butylation of the cyclic tetramer was carried out as previously reported [63,64].

The product was characterized by ^1^H NMR at 298 K to give the following signals: 

^1^H NMR (500 MHz, CDCl_3_, *δ* in ppm); 10.19 (s, OH, 4 H (**1**)); 7.04 (d, Ar-H, 8 H (**3**)); 6.7 (t, Ar-H, 4 H (**2** & **2′**)); 4.26 (d, H-axial, 4 H (**4**)); 3.48 (d, H-equatorial, 4 H (**4′**)).

#### 3.1.2. Preparation of 5,11,17,23-tetra[(4-ethylacetoxyphenyl) (azo)]calix[4]arene, CA-AZ

**Scheme 1:**
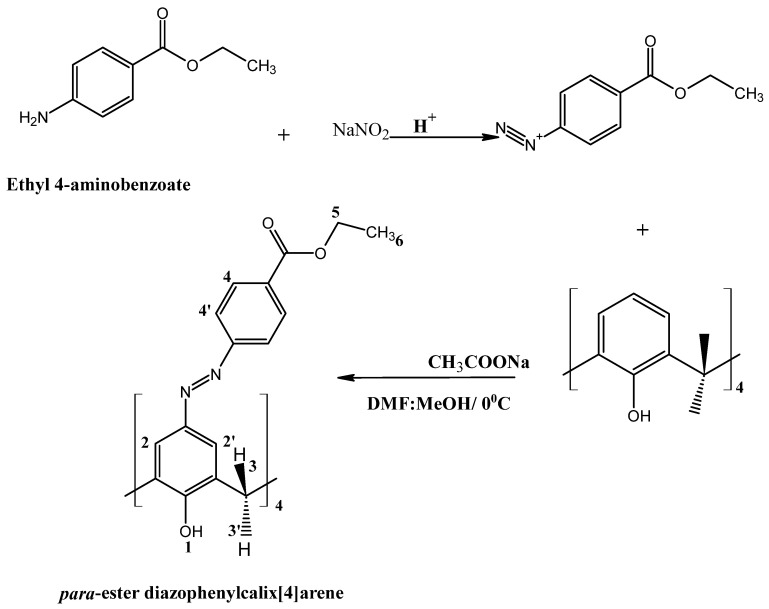
Synthetic procedure used for the preparation of CA-AZ.

The compound was synthesized by a diazotization reaction [65] described in Scheme 1.

In a 500 cm^3^ round-bottomed flask, ethyl 4-aminobenzoate (1.29 g, 7.8 mmol), sodium nitrite (0.41 g, 6.00 mmol) and conc. HCl (14 cm^3^) in water (25 cm^3^) were added gradually to a cold solution (0–5 °C) of 25, 26, 27, 28-tertrahydroxy calix[4]arene (0.64 g, 1.5 mmol) and sodium ethanoate trihydrate (1.17 g, 8.6 mmol) in a DMF/MeOH (2:1) mixture to obtain a dark-orange-coloured suspension. The mixture was stirred for 6 h; a red precipitate was observed. The mixture was filtered, and the residue was washed with cold water and then methanol several times. The product was left on the Schlenk line for one week (0.63 g, 98% yield).

The product was characterized by ^1^H NMR (500 MHz) and ^13^C NMR (100 MZ) at 298 K. 

^1^H NMR (500 MHz, CDCl_3_, *δ* in ppm); 10.25 (s, OH, 4 H **(1)**); 8.13 &8.14 (d, Ar-H, 8 H (**4** & **4′**)); 7.85 (s, Ar-H, 4 H (**2** & **2′**)); 4.39 (q, COO-CH_2_-CH_3_, 8 H (**5**)); 4.4 (d, H-axial, 4 H (**3**)); 3.87 (d, H-equatorial, 4 H (**3′**)); 1.4 (t, CH_3_, 12 H (**6**)). ^13^C NMR (100 MHz, CDCl_3_, 298 K, TMS): δ (ppm): 166; 155.1; 151.8; 147.8; 131.8; 130.5; 128.3; 124.8; 122.3; 61.2; 31.8; 14.3. 

Elemental analysis was carried out in duplicate at the University of Surrey; (C_64_H_56_N_8_O_12_) MW. (1129.20); Calculated %; C, 68.08; H, 5.0; N, 9.92. Found %; C, 68.14; H, 4.93; N, 10.2.

### 3.2. FT-IR, Thermogravimetric and SEM-EDX Analyses of CA-AZ and CA-AZ-F^−^ Salt

Compound (CA-AZ) and fluoride loaded CA-AZ were analysed via Fourier Transform Infrared Spectroscopy (using an Agilent Cary 600 Series FT-IR spectrometer). Measurements were made within the 4000–600 cm^−1^ range. Calibration spectra were made from 32 scans. The FT-IR spectra were scanned against air background spectrum. For CA-AZ thermal stability, analysis were recorded using a thermogravimetric analyser (TGA Q500 V6.7) under nitrogen gas. Sample was heated from 25 °C to 800 °C at a heating rate of 10 °C/min. 

Regarding SEM-EDX measurements, a scanning electron microscope (SEM) JEOL JSM-7100F, equipped with secondary and backscattered imaging coupled with Ultradry energy dispersive X-ray (EDX) for elemental analysis, was used for morphological investigations. CA-AZ treated with fluoride ion samples were mounted on an aluminium stub. Working conditions were 15 KeV for accelerating voltage and 10 mm for the detector working distance.

#### H NMR Complexation Studies

^1^H NMR complexation experiments were conducted at 298 K by dissolving the ligand (2.17 × 10^−4^ mol·dm^−3^) in DMSO-d_6_ and then adding a known amount of the salt (1.10 × 10^−3^ mol·dm^−3^) into an NMR tube using TMS as an internal standard. Chemical shift changes (Δ*δ*, ppm) were calculated by subtracting the chemical shift of the free ligand (reference spectrum) from the chemical shift of the complex. Chemical shift changes (Δ*δ*) values greater than 0.1 ppm were an indication of an interaction that requires further investigations.

### 3.3. UV-Visible Studies of CA-AZ and Ion Salts in Dimethyl Sulfoxide

UV-Visible studies were carried out using the Thermo Scientific Evolution 220 UV-Visible Spectrophotometer. Data were processed with INSIGHT ^TM^ 2 software. Quartz cuvette cell was used for sample measurement. Absorbances were recorded over a wavelength range of 350–750 nm. 

The selectivity of the synthesized calix[4]arene derivative along with its chromogenic properties were investigated using stock solutions of the ligand (1 × 10^−5^ mol·dm^−3^) and the anion salt (CO_3_^2−^, F^−^, H_2_PO_4_^−^, HSO_4_^−^ & Cl^−^) (1 × 10^−4^ mol·dm^−3^), (ammonium and potassium as counter-ions for carbonate and tetra-n-butyl ammonium as counter-ion for other anions) in dimethyl sulfoxide. Screening experiments on different cation and anion salt solutions were carried out prior to this step. A CA-AZ solution (3 cm^3^) was placed in quartz cell, and the absorbance spectrum was recorded against DMSO. UV-absorption measurements of the complex (ligand- ion salt) were also conducted in a solution (3 cm^3^) containing the same concentration of the ligand with the desired amount of salt solutions.

### 3.4. ^1^H NMR Titration Studies

^1^H NMR titration of *para*-ester diazophenylcalix[4]arene with F^−^ was carried out. The reference sample (0.5 cm^3^, 1 × 10^−4^ mol·dm^−3^) was prepared in deuterated dimethyl sulfoxide, and another solution of the anion salt was made at a concentration of 2 × 10^−4^ mol·dm^−3^ in 2 cm^−3^ of the same solvent. ^1^H NMR measurements were performed for the reference sample, then aliquots of the anion salt were added stepwise (9 additions for each anion of 40 μL), and measurements were carried out following each addition. Chemical shift changes (Δ*δ*) were calculated.

### 3.5. Conductometric Titrations of Para-Ester Diazophenylcalix[4]arene and Fluoride Anion in Dimethyl Sulfoxide at 298.15 K

For conductometric titration experiments, a solution of fluoride ion salt in dimethyl sulfoxide (25 cm^3^) was prepared and transferred to the conductometric cell. Then, the electrodes were inserted, and the solution was stirred for a few minutes until thermal equilibrium was established. Afterwards, the ligand solution prepared in the same solvent was added stepwise until stable readings were observed. The reciprocal of resistance, L (Ω^−1^) was calculated following each addition. The molar conductance was then calculated as previously [19].

### 3.6. Calorimetric Titration Experiments

Calorimetric titration experiments (direct and competitive) were carried out in dry dimethyl sulfoxide at 298.15 K as previously described [66]. 

### 3.7. Carbon Dioxide Detection studies by Para-Ester Diazophenylcalix[4]arene-Fluoride Complex Using FTIR

FTIR was the analytical technique used in this section to determine the sensitivity and complexation of the carbon dioxide gas with solid *para*-ester diazophenylcalix[4]arene-fluoride complex.

The solid complex salt was prepared in a film in THF solvent. Then, the solvent was evaporated, and the complex salt was exposed to carbon dioxide where FT-ATR measurements were made. The spectrum was recorded as described in Section 3.3.

#### Extraction of CO_2_ by the Solid Complex Salt

A sample of the solid complex salt (1.2 g) was deposited on a quartz wool where it was placed in a tubular fixed-bed quartz reactor. The experiment was performed at atmospheric pressure, and the temperature was controlled by a thermocouple. The outlet gas (CO_2_) was monitored using infrared and thermal conductivity detectors on an ABB AO2020 online gas analyser. The outlet total volumetric rate of the gas was measured with a flow meter. 

## 4. Conclusions

From the above discussion, the following conclusions are drawn:

(i) A new upper rim azo calix[4]arene derivative has been synthesized and fully characterized.
(ii) ^1^H NMR studies in DMSO-d_6_ show the conformational changes that the receptor undergoes as a result of the medium effect and anion complexation processes.
(iii) The thermodynamic parameters of complexation (log K_s_, $\Delta_{c}G^{o}$, $\Delta_{c\;}H^{o}$ and $\Delta_{c\;}S^{o}$) have been determined using titration calorimetry. Among the univalent anions, a quantitative measure of the stability of the complexes (log K_s_) shows that the receptor is selective for fluoride relative to other 1:1 univalent anions. However, the higher selectivity of the receptor for the carbonate anion relative to fluoride or any other anion is not an issue given that salts other than fluoride are unable to interact with this gas in the air.
(iv) The mode of interaction of the receptor with the anion was established and consisted of the deprotonation of one of the phenolic units in the narrow rim of the receptor followed by the protonation of the functionalized upper rim which provided the sites of interaction with the anion.
(v) The ability of the fully characterized anion salt to remove CO_2_ from the air is illustrated through the text, together with the recycling of the complex salt. This is a very important aspect to highlight given that, so far, most reports on calixarene chemistry are centred on the complexation process. This paper calls upon the need to explore further the practical applications of macrocyclic complex salts.
(vi) Our approach based on a calix[4]arene fluoride salt for sensing and capturing CO_2_ has several advantages relative to using calixarene derivatives attached to silica [48] in that (i) the synthetic procedure is simpler, (ii) the kinetics of the process is very fast, (iii) the CA-AZ interaction with CO_2_ results in a colour change which can be easily detected by the naked eye, and (iv) the receptor can be easily regenerated.

## Figures and Tables

**Table 1:** ^1^H NMR data for CA-AZ in deuterated solvents at 298 K (DMSO was selected as a reference solvent).

** 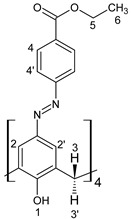 **								
***δ*** **(ppm)**								
Solvent	H-1	H-2,2′	H-3 H-Equatorial	H-3′ H-Axial	H-4	H-4′	H-5	H-6
CDCl_3_	10.25	7.85	3.87	4.40	8.13	8.14	4.39	1.4
DMSO-d_6_	-	7.82	3.51	4.34	7.85	8.05	4.31	1.32
∆δ	-	+0.03	+0.36	+0.06	+0.28	+0.09	+0.08	+0.08

[CA-AZ] = 2.17 × 10^−4^ mol·dm^−3^; V = 0.5 cm^3^.

**Table 2:** ^1^H NMR chemical shift changes (∆δ, ppm) resulting from addition of anion salts (as tetra-n-butyl ammonium) to CA-AZ relative to the free ligand in DMSO-d_6_ at 298 K.

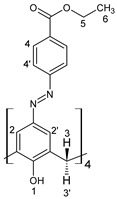								
***δ*** **(ppm)**								
	H-1	H-2	H-3	H-3′	H-4	H-4′	H-5	H-6
*δ*_Ref_	-	7.82	3.51	4.34	7.85	8.05	4.3	1.32
F^−^	-	-	−0.19	−0.64	-	-	-	−0.39
CO_3_^2−^	-	−0.2	0.12	0.28	−0.1	−0.08	0.0	−0.01
H_2_PO_4_^−^	-	−0.17	-	0.3	−0.09	−0.06	0.0	−0.39
HSO_4_^−^	-	−0.01	−0.18	0.6	0.0	0.0	0.0	−0.39
Cl^−^	-	0.02	0.17	0.08	0.0	0.0	−0.08	−0.39

[X^−^] = 1.10 × 10^−3^ mol·dm^−3^; V = 0.5 cm^3^; [CA-AZ]= 2.17 × 10^−4^ mol·dm^−3^; V = 0.5 cm^3^.

**Table 3:** Thermodynamic parameters of complexation of *para*-ester diazophenylcalix[4]arene and anions in DMSO at 298.15 K.

Anion	CA-AZ: X^n−^	Log K_s_	$\Delta_{c}G^{o}\;(\mathrm{kJ}\;{\mathrm{mol}}^{-1})$	$\Delta_{c}H^{o}\;(\mathrm{kJ}\;{\mathrm{mol}}^{-1})$	$\Delta_{c}S^{o}\;(J\;{\mathrm{mol}}^{-1}\;K^{-1})$
Cl^− a^	1:1	-	-	0.2 ± 0.3	-
F^− b^	1:1	5.7 ± 0.1	−32.6 ± 0.2	−14.6 ± 0.5	60
CO_3_^2− a^	1:1	6.72 ± 0.05	−38.4 ± 0.1	−29.8 ± 0.3	29
	1:2	4.3 ± 0.05	−25.5 ± 0.1	14.2 ± 0.3	130
	(1 + 2)	11.02 ± 0.05	−63.9 ± 0.1	−15.6 ± 0.3	159
HSO_4_^− a^	1:1	2.5 ± 0.2	14.4 ± 0.5	7.2 ± 0.3	73

^a^ Direct calorimetric titration, ^b^ Competitive calorimetric titration.

## Data Availability

Not applicable.
